# PROTOCOL: Involving men and boys in family planning: A systematic review of the effective components and characteristics of complex interventions in low‐ and middle‐income countries

**DOI:** 10.1002/cl2.1140

**Published:** 2021-01-19

**Authors:** Áine Aventin, Martin Robinson, Jennifer Hanratty, Eimear Ruane‐McAteer, Mark Tomlinson, Mike Clarke, Friday Okonofua, Chris Bonell, Maria Lohan

**Affiliations:** ^1^ Queen's University Belfast Belfast UK; ^2^ University College Cork Cork Ireland; ^3^ Stellenbosch University Stellenbosch South Africa; ^4^ Women's Health Action and Research Centre Benin City Nigeria; ^5^ London School of Hygiene and Tropical Medicine London UK

## 1 BACKGROUND

### 1.1 The problem

Family planning (FP) helps people avoid unintended pregnancy, attain their desired number of children and/or determine the spacing of pregnancies. Effective FP is achieved through the use of contraceptive methods, provision of safe abortion, and prevention and treatment of infertility. FP also contributes to reduced maternal, neonatal and child morbidity and mortality, as well as the negative economic and psychosocial implications that unintended pregnancy, pregnancy complications and infertility can have.

Despite determined progress since the implementation of the United Nations' Sustainable Development Goals (SDGs; United Nations, 2015), reports indicate that progress has been slower than expected in relation to maternal and child health and gender equality (FP2020, 2018; UNICEF, 2018; World Health Organisation, 2017). If current trends continue, more than 50 low‐ and middle‐income countries (LMICs) will not meet their SDG under‐five mortality target by 2030 and 56 million children under age‐5 will die (UNICEF, 2018). Equally, achieving the SDG target of a global maternal mortality rate of below 70 per 100,000 births will require a reduction in current rates of an average of 7.5% each year until 2030. This is more than three times the current 2.3% annual global rate of reduction (World Health Organisation, 2016). At the current rate of change, it will take 200 years (nine generations) to reach the SDG 5 goal of achieving gender equality and empowering women and girls (Organisation for Economic Co‐operation and Development, 2019). Further, by 2018, only 46 of FP2020′s targeted 120 million additional women using contraception had been reached—a clear indicator of the work that remains to be done in order to reach the 2030 SDGs (FP2020, 2018).

Every year, around 300,000 women and girls die during childbirth or from pregnancy‐related complications, including unsafe abortion, with the vast majority of these deaths (94%) occurring in LMICs (World Health Organisation, 2019). Equally, unintended and mistimed pregnancies also contribute to the burden of high infant morbidity and mortality (Kozuki et al., 2013; Say et al., 2014; Singh et al., 2013). Around 2.7 million new‐borns die every year in LMICs and many more suffer from disease relating to preterm birth, being small for gestational age and malnutrition (Guttmacher Institiute, 2018). Provision of evidence‐based interventions to accelerate the use of FP is, therefore, a matter of life and death for people in LMICs. Despite declines in global fertility rates, unmet FP needs remain high. An estimated 214 million women in LMICs would like to avoid or delay pregnancy, but are not using contraception (Guttmacher Institiute, 2018). There is, therefore, an urgent need to understand how to accelerate the use and impact of FP programmes.

Involving men and boys in FP is now recognised as essential for optimising positive maternal and child health outcomes (Croce‐Galis et al., 2014; Hardee et al., 2017; Lohan et al., 2017; Phiri et al., 2015). Male involvement in FP has been associated with increased uptake of FP services, HIV counselling and testing reduction in risk behaviours, improved maternal health and spousal communication (Nkwonta & Messias, 2019). Further, FP programmes that adopt a focus on transforming gender inequalities show particular promise (Phiri et al., 2015). The underpinning logic behind involving men in FP recognises that, in many countries, men are the primary decision‐makers on family size, birth spacing, and their partners use of FP and also that uptake of contraception among men themselves is insufficient (Nzioka, 2002). Research has shown that a lack of decision‐making power among women can impact negatively on attempts to improve reproductive health including uptake of FP, breastfeeding and cervical cancer screening (Nkwonta & Messias, 2019). International health and development frameworks therefore emphasise the importance of working with both males and females in order to improve uptake of FP and sexual and reproductive health (SRH) outcomes for all (Group, 2015; WHO, 2011).

In practice, “involving” men and boys in FP can range from encouraging men to be supporters of autonomous FP decision‐making among women to more expansive conceptualisations of men as both supporters and users of contraceptive methods, leading change in relation to FP uptake in their families and communities as well as meeting their own reproductive health needs (Hardee et al., 2017; Lohan, 2015). Intervention activities can range from couple counselling and individual invitations from SRH services to media campaigns (Nkwonta & Messias, 2019). *Gender Transformative* (GT) approaches to male involvement in SRH aim to change harmful gender and power imbalances and encourage women's autonomy in sexual decision‐making (Interagency Gender Working, 2017; Kagesten & Chandra‐Mouli, 2020). According to the World Health Organisation (WHO) definition, a GT approach “seeks to challenge gender inequality by transforming harmful gender norms, roles and relations through programmatic inclusion of strategies to foster progressive changes in power relationships between women and men” (Ruane‐McAteer et al., 2019; World Health Organisation, 2011). Programme planners now also understand that, in order to be truly transformative, FP interventions involving men and boys must also seek to address the intersectional influences of other social factors on gender inequalities including race, ethnicity, sexual orientation, and poverty (Kagesten & Chandra‐Mouli, 2020; Kågesten et al., 2016).

A recent WHO review of reviews (Ruane‐McAteer et al., 2018) and evidence and gap map (EGM; http://srhr.org/masculinities/rhoutcomes/) conducted by members of our team revealed, however, that there are few systematic reviews of the characteristics and components of effective programmes that involve men and boys in FP and none that attempt to identify the causal chain mechanisms that lead to successful outcomes, and which take account of individual‐ and system‐level moderators as well as process‐level barriers and facilitators. This paucity of review evidence means it remains unclear whether existing interventions are fit for purpose or suitable for scale‐up across different contexts and populations.

### 1.2 The Intervention

This review will include any behavioural and service‐level interventions aiming to improve the uptake of FP by directly involving men or boys, either in isolation or alongside women and girls, in LMICs. As noted above, the focus on men and boys in LMICs reflects the concerted movement toward male involvement in FP programming as a potentially effective method of achieving improved health outcomes for all, especially in contexts such as LMICs where the unmet need for FP is greatest (Hardee et al., 2017; Phiri et al., 2015; World Health Organisation, 2011). The focus on men and boys also recognises the importance of examining the impact of addressing gender inequalities in FP programming and engaging men as *both supporters and users* of FP and not just supporting actors in contraceptive uptake for their female partners (Hardee et al., 2017). Consideration of eligible interventions for this review was informed by the following:

1. Reference to the details of 61 FP interventions that were included in a 2018 WHO EGM of SRHR Interventions involving men and boys conducted by members of the team;
2. Reference to findings of a Rapid Review of 63 FP intervention studies involving men and boys in LMICs, conducted as part of the current study which indicated a broad range of intervention characteristics, theoretical frameworks and outcomes; and
3. Consultation with our international advisory group of more than 30 experts in FP and SRHR and project consultants who reviewed our drafted list of eligible interventions and provided feedback based on their extensive experience of the subject.

Eligible interventions will include those that aim to increase the uptake of FP (male and/or female contraception; safe abortion and safe postabortion care) aiming to ensure:

- Decreased unmet need for FP;
- Avoidance of unintended or unwanted pregnancies;
- Birth spacing (i.e., choice in relation to time period between pregnancies);
- Birth limiting (i.e., choice in relation to limiting family size).

While FP methods also include medical, surgical and behavioural (lifestyle) interventions for addressing *infertility*, we will not examine these in the current review. The majority of fertility‐focused interventions are medical or surgical in nature (Ruane‐McAteer et al., 2019), and those that target behavioural determinants are generally focused on lifestyle changes such as reducing smoking and obesity and increasing exercise (Lan et al., 2017). In consultation with our international expert advisory group, we agreed that because the theoretical basis, components, and characteristics of such interventions differ greatly from those aiming to prevent unintended pregnancy, they were outside the scope of the current study. However, should an included study address infertility alongside any of the above outcomes, that study will be included assuming it meets our other inclusion/exclusion criteria.

We expect that interventions will include those delivered in education, health or community settings aiming to increase capability (knowledge, skills), opportunity (access, social support) and motivation (attitudes, norms) to use FP methods via mass, small or social media information, face‐to‐face communication; health service enhancements; monetary and other incentives; and access to FP methods.

Intervention components and activities may include, but are not limited to, a combination of some or all of those identified in our ongoing rapid review of theories and outcomes of FP interventions involving men and boys (Robinson et al., 2020) and consultation with the more than 30 members of our international expert advisory group:

- *Gender dialogue* (addressing gender inequalities and harmful/restrictive gender norms);
- *Information provision* (in clinics, educational settings, community settings, comprehensive sex education);
- *Skills‐ building* (workshops, demonstrations, modelling, enablement);
- *Problem‐solving* (identifying barriers and facilitators of FP communication and access; supporting autonomous decision making);
- *Social support* (outreach with male motivators, mentors, peer support, engaging religious leaders, community dialogue, reinforcement);
- *Incentivisation* (e.g., conditional cash transfer, vouchers);
- *Mass Communication* (social marketing, mass media, social media, mHealth, hotlines); and
- *Health service enhancement* (low‐cost/free access to FP methods and services; health service adaptations).

As indicated by a further WHO systematic review of interventions involving men and boys across all WHO SRH and rights outcomes (Ruane‐McAteer et al., 2019), and from consultation with our advisory group, we expect that eligible interventions will include, but not be limited to, those that vary by:

- *Rationale* or *goal* (e.g., contraceptive uptake and/or addressing unequal gender norms);
- *Theoretical approach* (e.g., behaviour change theory; gender theory);
- *Approach to intervention design* (e.g., codesign or coproduction);
- *Materials and procedures* (including approach to engaging men and type of contraceptive method);
- *Who provides* (e.g., health or education professionals, peers, trained facilitators);
- *Who receives* (e.g., adolescents/youth/adults; males only; males and females);
- Modes of delivery (e.g., face‐to‐face, online; individuals/couples/community);
- *Delivery setting* (e.g., home, community, educational);
- *Dose and intensity* (how much, how often, how long); and
- *Tailoring, modifications, adherence, or fidelity*.

Of particular relevance to this review, we expect that eligible interventions will vary according to whether or not they address unequal gender norms in FP. The modification of gender norms can be categorised on a continuum from “gender‐unequal/neutral” approaches which reinforce or ignore unequal norms, roles and relations, thereby perpetuating gender‐based discrimination; to “gender‐sensitive/specific” approaches, which do consider gender norms, roles and relations and/or men and women's specific needs or roles but do not seek to change gender inequalities; to “gender transformative” approaches which are inclusive of gender‐sensitive and gender‐specific strategies, but also challenge gender inequalities by transforming harmful gender norms, roles and relations through programmatic strategies that foster progressive changes in power relationships between women and men (Interagency Gender Working Group, 2017; World Health Organisation, 2011). While it is possible that it may be unclear where interventions lie in relation to this continuum, we will endeavour to categorise interventions accordingly and report instances in which categorisation is not possible.

Finally, based on findings from our ongoing rapid review (Robinson et al., 2020) and consultation with our advisory group experts, we expect that eligible studies will present a variety of individual‐ and system‐level moderators of interest. These may include, but not be limited to:

*Individual level moderators*

- Age/life stage
- Sex
- Ethnicity
- Disability
- Sexual orientation
- Gender identity
- Religion/religiosity
- HIV/AIDS/STI status
- Marital status/history/type
- Relationship status
- Reproductive history
- Sex of existing children
- Past FP behaviours and experiences
- Coresidence with children, partner, extended family
- Urban/rural residence
- Migrant status
- Attitudes values and beliefs about FP
- Perceived gender and cultural norms
- Attitudes about sexual pleasure
- Socioeconomic factors (e.g., student, employed, unemployed; poverty; income level; education level)

*External factors/system level moderators*

- Social norms (gender, cultural, religious)
- Political and economic climate
- Legal and historical context
- Health policies and strategies
- Health systems and availability of services
- FP supply and provider characteristics
- Delivery setting characteristics and policies
- Conflict/disaster/disease/climate‐stress factors

## 2 HOW THE INTERVENTION MIGHT WORK

**Figure 1:**
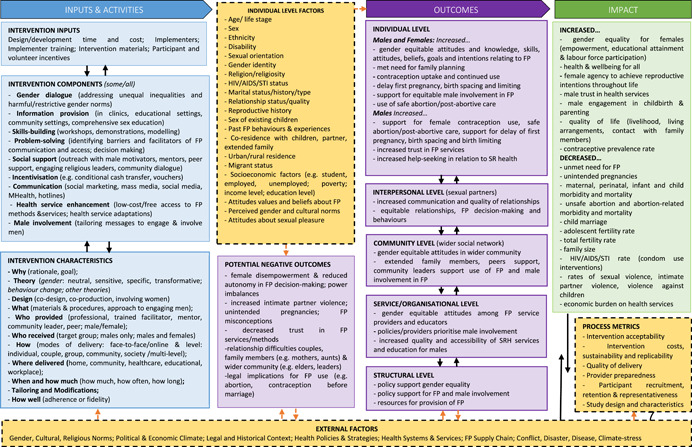
*INVOLVE_FP* Logic model for a review of complex behavioural interventions involving men and boys in low‐ and middle‐income countries in family planning

This review will examine existing knowledge from quantitative and qualitative research on interventions involving men and boys in FP in LMICs. The aim is to deepen our understanding of the dynamics of these interventions and allow us to provide recommendations for future research and the optimal use of evidence by decision makers, FP practitioners and intervention programmers. Using a Causal Chain Analysis (CCA) approach (Kneale et al., 2018), we will use the logic model presented in Figure 1 to frame both data extraction and subsequent CCA of intervention characteristics and outcomes.

The logic model was built based on: (a) the research team's own expertise, drawing on evidence on achieving desired family size identified in our previous WHO reviews (Ruane‐McAteer et al., 2019; Ruane‐McAteer et al., 2020); (b) a rapid review of theories used in FP interventions involving men and boys (Robinson et al., 2020); and (c) consultation with our expert advisory group. It provides a visual representation of how and under what circumstances, FP interventions might work to increase uptake of FP, help people attain their desired family size and ultimately result in improvements in maternal and child health, gender equality, SRH and rights, quality of life and improved livelihoods for all. The logic model is informed by feminism and feminist‐informed masculinity studies (Greene & Biddlecom, 2000; Lohan, 2015; Marsiglio et al., 2013; Van der Gaag, 2014), as well as social‐ecological theories of behaviour (Bronfenbrenner, 1992), psychosocial theories of behaviour change (Atkins et al., 2017; Bandura, 1986), and realist interpretations of causality (Pawson et al., 2005). It sets out the multiple possible pathways through which each intervention component, or combination of components, would bring about positive outcomes and changes at the individual, interpersonal, community, organisational, and structural levels. In essence, we hypothesise that in order to positively impact maternal and child mortality and morbidity indicators, FP interventions involving men and boys first need to effect change in one or more outcomes at proximal (individual), intermediate (interpersonal, community, organisational/service) and distal (structural) levels. Programmes will, however, be eligible for inclusion if they measure only proximal outcomes. As illustrated in the model, changes in these outcomes follows from exposure to an intervention, although different combinations of intervention characteristics are possible and may have differential impact, and may also be influenced by the characteristics of the participants and the context in which the intervention takes place. Each FP intervention will include core components as well as a set of resources and theory underlying its implementation. Further, the logic model recognises that interventions can fail to produce change because of issues relating to design or implementation processes (e.g., the intervention may not be well implemented, implementation may not trigger mechanisms or mechanisms may not generate outcomes) and, therefore, incorporates ways of understanding the success of the implementation. It also recognises that potential negative outcomes are possible for every intervention, and incorporates potential indicators of these.

The logic model will be used as the foundation for the evidence synthesis, informing decisions at all stages of the review process. This approach addresses a common criticism of systematic reviews and meta‐analysis (that they are limited to providing basic conclusions regarding effectiveness) and moves toward a more nuanced identification of what works, for whom and under what circumstances (Pawson et al., 2005). Using a CCA approach will allow the examination of the active ingredients of effective interventions, testing of causal pathways, and identification of system‐ and process‐level barriers and facilitators to effective intervention. Our synthesis will enable evaluation practitioners and service providers to modify and optimise existing FP interventions to maximise efficacy in accelerating FP use and adaptation for use in different settings.

## 3 WHY IT IS IMPORTANT TO DO THIS REVIEW

### 3.1 Existing reviews

A recent WHO EGM completed by members of our team (Ruane‐McAteer et al., 2019) found 146 existing systematic reviews of studies involving men and boys in FP.^1^ Given that FP is a broad concept, including everything from the prevention of unintended pregnancies to treating infertility, the number of reviews identified is unsurprising. However, among those reviews that do exist, 85 concern medical interventions for the treatment of male infertility and 61 address behaviour‐change and service‐level interventions to promote behaviour change in FP.

Examination of these 61 reviews revealed that they differ from the current review in a number of ways: 15 included only interventions conducted in high‐income countries; 28 were limited to populations on the basis of age (i.e., only young people or adults and not inclusive of both); and 34 focused on intervention effectiveness only. We identified 28 reviews that examined the components and characteristics of FP interventions. However, only three of these reported the characteristics and approaches associated with intervention effectiveness alongside an examination of causal processes.

Of these three reviews that examined causal processes, we identified further differences that justify the need for this review. Phiri et al. (2015) examined the role of behaviour change techniques in six randomised trials to promote modern contraceptive uptake in LMICs, finding that those that involve male partners to be most effective. The evidence presented was, however, summarised and analysed narratively for the association between behaviour change techniques (programme inputs) and positive behaviour change (e.g., contraception uptake and use).

A second review examined the use of behaviour change theories and a gender integrated programming approach to affect health‐related behaviours (Schriver et al., 2017). This review included quantitative and qualitative studies to inform a narrative synthesis and focused primarily on the effects of GT programming. While this review did encompass FP and contraceptive use interventions, these were not the only health behaviour outcomes under investigation. Our proposed review differs from this in that we will examine the myriad of programming approaches that involve men and boys (i.e., not only GT approaches) and how these specifically effect change in FP behaviours.

Finally, Lopez et al. (2009) examined the behaviour change theories underpinning interventions for contraceptive uptake and their association with positive behaviour change. The review also made use of narrative synthesis to present results, noting that theory‐based interventions were associated with more positive outcomes. The authors describe how programmes based on a theory of change provide a framework to explain how change is affected, however, they did not seek to analyze the proposed processes.

Outside of reviews featured in the EGM, an earlier review of 63 studies published between 1995 and 2008 conducted by Mwaikambo et al. (2013) was also identified. This review sought to examine strategies associated with positive change in FP interventions. These studies were, however, quantitative evaluations of effectiveness and lacked qualitative data on processes. This review did not attempt to conduct meta‐analysis or re‐analysis of programme evaluations, instead presenting a narrative synthesis of intervention strategies and characteristics associated with positive FP outcomes. Further, Mwaikambo et al. (2013) noted the relevance of “male involvement” as a potentially effective programme strategy and limitations of the available evidence in their review for this.

Members of our team have also recently completed a systematic review of GT interventions with men and boys, as derived from the reviews identified by the EGM (Ruane‐McAteer et al., 2019). Unlike the proposed review, this systematic review (Ruane‐McAteer et al., 2020) focused on understanding the characteristics of effective GT interventions across all WHO defined SRH and rights outcome domains. The analysis was also a narrative synthesis of the effective characteristics of GT interventions rather than a quantitative analysis of causal mechanisms between programme inputs and intended outcomes.

Searches of Campbell, Cochrane and PROSPERO databases indicated that most ongoing FP‐focused reviews are limited to female outcomes and none use CCA or focus on understanding mechanisms of change.

This current review will encompass data on multiple variables that may influence FP and is, therefore, complex. It will make a unique contribution by providing an updated search of the literature on FP interventions that involve men and boys and by examining mechanisms of change in FP interventions involving men and boys using novel methods of analysis. Working with stakeholders from LMICs, integrating both qualitative and quantitative research, and using CCA methods to frame the review and inform synthesis decisions, we will assess the strength of evidence in the area, and uncover the key components and critical process‐ and system‐level characteristics of successful interventions. Despite extensive searches, we have not identified any existing or ongoing reviews that employ these methods or have this scope.

### 3.2 Relevance of the review findings to policy and practice

The proposed evidence synthesis addresses priority health challenges and outcomes that are directly relevant to global development policy. Using a rationale and methodology underpinned by goals set forth by the 2030 SDGs 3 and 5 (United Nations, 2015), the review seeks to synthesise evidence from multiple countries, disciplines and stakeholders in order to develop globally relevant solutions to challenges relating to maternal and child health (SDG 3.1 and 3.2), gender equality, and the empowerment of women and girls (SDG 5.6 and 5.9). The proposed outcomes also relate directly to the WHO's Reproductive Health Strategy (World Health Organisation, 2004).

The review will directly involve expert stakeholders from across the world in a study advisory group, helping ensure that the findings will be relevant where they are needed most. Further, the review will use innovative synthesis methods while also producing useful findings. As well as addressing the gap in knowledge resulting from the lack of review evidence relating to the characteristics of FP interventions that involve men and boys, it will act as an exemplar for evaluation practitioners wishing to use CCA to conduct systematic reviews of complex interventions. It will be of value to both FP policy makers and practitioners in LMICs because it will produce easy‐to‐access recommendations for practice directly relevant to their work “on the ground.”

As such, we anticipate that the synthesis would be of relevance to: (a) programme developers and evaluators conducting FP research in LMICs; (b) national and international development organisations including DFID, WHO, UNICEF, UNESCO, OCED, UNFPA, UNWOMEN and The World Bank; (c) global SRH and FP service providers and partnerships such as The International Federation for Planned Parenthood and Family Planning 2020 (FP2020); and (d) decision‐makers at Ministries of Health, Medical Research Councils/Institutes for Medical Research and local FP service providers in the nine participating countries as well as other LMICs seeking to accelerate the use of FP.

## 4 OBJECTIVES

The aim of the review is to uncover the mechanisms of change in FP interventions involving men and boys. While it is now recognised that FP interventions involving men and boys have better outcomes than those that do not involve men and boys (Croce‐Galis et al., 2014; Hardee et al., 2017; Lohan et al., 2017; Phiri et al., 2015), less is known about the underlying mechanisms and causal pathways. Working with an international expert advisory group and using CCA methods to frame the review and inform synthesis decisions, we will assess the strength of evidence in the area, and uncover the key components and critical process‐ and system‐level characteristics of successful interventions. Building and testing a logic model as part of the process, the review will seek to confirm or refute theories about how involving men and boys in FP programmes in LMICs can impact on health outcomes. In this way, it will enable better understanding of the suitability of existing interventions for adaptation and scale‐up. The following review questions were developed in consultation with our international advisory group:

1) What is the nature and extent of experimental evidence on engaging men and boys in FP and what gaps in research knowledge exist?
2) What are the impacts of FP interventions involving men and boys on FP‐related outcomes?
3) What are the effective components of interventions that achieve positive change in intended FP outcomes?
4) What characteristics and combinations of characteristics are associated with positive FP‐related outcomes?
5) Do outcomes vary by context and participant characteristics?
6) Are there any unintended or adverse outcomes?
7) What are the system‐ and process‐level barriers to and enablers of effective models of FP involving men and boys?

## 5 METHODS

### 5.1 Criteria for considering studies for this review

#### 5.1.1 Types of studies

Randomised trials (individual or cluster), quasiexperimental studies (including quasirandomised trials, pre‐ and posttest with control group and other relevant designs such as interrupted time series studies) and, where available, their associated qualitative/mixed methods studies (e.g., formative qualitative research, process evaluations, and qualitative research exploring accounts of how the interventions work). Mixed methods evaluations will similarly be eligible for inclusion where the quantitative design satisfies the criteria mentioned above.

#### 5.1.2 Types of participants

Males over 10 years of age of any sexual orientation and gender identity. While we will consider outcomes for both men and women, the population that receives the intervention must include men or boys. Interventions or studies with women and girls only are not eligible.

#### 5.1.3 Types of interventions

Behavioural and service‐level interventions, directly targeting or involving men or boys in LMICs, that aim to improve uptake of FP methods. The interventions in included studies will be categorised using a taxonomy that builds on the list provided under “intervention” above but will be developed inductively based on the intervention descriptions provided in the studies.

*Setting*

Health, education and community settings in LMICs.

*Comparisons*

- Alternative intervention
- Usual/standard care
- No intervention
- Attention control

#### 5.1.4 Types of outcome measures

The relevant outcomes for this review were chosen through part of the stakeholder‐informed logic model development phase of this study. In the logic model we illustrate proximal and distal outcomes that relate to maternal and child health and FP‐related outcomes. We recognise that some outcomes featured in the review logic model, such as community, organisational and structural level outcomes and distal impacts, may not have been measured in the studies eligible for inclusion in this review but we will examine any combination of outcomes provided. Further, while we include met need for FP as a key rights‐based outcome, we include other outcomes in recognition that not all interventions take a rights‐based approach. The *Primary* and *Secondary* outcomes of interest in the current review are as follows:

##### Primary outcomes

1. *Met need for FP* (e.g., decreased unmet need for FP, increased met need for FP).
2. *Gender equitable attitudes and behaviours* (e.g., more positive gender norms, equitable FP decision making, decrease in male dominated FP decision making).
3. *Sexual and reproductive health behaviours* (e.g., contraception uptake, sustained use, use of more effective methods, reducing unprotected sex, decreasing age of sexual debut, abstinence, birth spacing, birth limiting).
4. *Family planning service use and engagement* (e.g., knowledge of FP services, frequency of use, support for partner engagement, use of safe abortion and/or postabortion care, increased trust in FP services, increased help‐seeking in relation to SRH more broadly).
5. *Fertility* (e.g., adolescent fertility rates, decrease in unintended pregnancy).

##### Secondary outcomes

6. *Psychosocial determinants of family planning behaviour* (e.g., knowledge, attitudes, skills, social norms).
7. *Relationship quality and discordance* (e.g., self‐rated relationship satisfaction, prevalence of intimate partner violence, increased couple communication).
8. *Attitudes toward FP services* (e.g., increased trust in FP services, increased help‐seeking in relation to SRH).
9. *Community level outcomes* (e.g., gender equitable attitudes in wider community, extended family members, peers support, community leaders support use of FP and male involvement in FP).
10. *Service/organisation level outcomes* (e.g., gender equitable attitudes among FP service providers and educators; policies/providers prioritise male involvement; increased quality and accessibility of SRH services and education for males).
11. *Structural level outcomes* (e.g., policy support for gender equality; policy support for FP and male involvement; resources for provision of FP).

As this review examines the causal chain of behaviour change, it is possible that these outcomes may feature with other intermediary outcomes that detail the processes of FP behaviours.

##### Duration of follow up

Where the same outcome construct is measured but across multiple time domains, such as through the collection of both posttest and further follow‐up data, we will seek to conduct and report the analysis separately for different time points at intervals of: <3 months, between 3 and 6 months, between 7 and 12 months, and over 12 months.

##### Types of settings

The focus of our research will be LMICs. As such, inclusion criteria will be limited to studies reporting interventions or programmes implemented in countries categorised as Low Income, Lower‐Middle Income, or Upper‐Middle Income by the World Bank (2019). Studies that report on multicountry interventions will be eligible if any one meets these criteria as an LMIC.

### 5.2 Search methods for identification of studies

As we will include both quantitative studies and qualitative studies, our search will have two phases. The first phase will be a comprehensive search for randomised trials and quasiexperimental studies. The second phase will be a search for qualitative studies limited to the specific experimental evaluation studies identified in phase one. Both searches will be conducted using the databases, grey literature sources and other approaches detailed below. Relevant qualitative studies may be identified in the first phase of the search and these will be retained for the second phase of the review. We anticipate that most qualitative studies will be found through forward citation searching.

a) *Databases*
  CINAHL, MEDLINE, PsycINFO, Social Science Citation Index–expanded, Cochrane Library (including CENTRAL), Campbell Systematic Reviews Journal, Embase, Scopus, Global Health Library (including African Index Medicus, Index Medicus for the Eastern Mediterranean Region, Index Medicus for the South‐Eat Asia Region, Latin America and the Caribbean Literature of Health, and Western Pacific Region Index Medicus) will be searched using relevant terms.
b) *Grey literature*
  Searches of Grey literature databases (ETHoS, ClinicalTrials.gov Register, ProQuest Dissertation & Thesis A&I, OpenGrey.eu, ELDIS.org) and searching of reports shared by relevant organisation websites (DFID, FP2020, United Nations Library/UNFPA, IPPF, 3ie, USAID, Promundo, FHI360, Population Council, Population Reference Bureau, Institute for Reproductive Health, Marie Stopes). We will also conduct internet searches using keywords in Google and scanning the first two pages of results for each keyword combination.
c) *Other approaches*
  Members of the International Expert Advisory Group will be asked to highlight any potentially relevant published or unpublished literature they are aware of related to the objectives of this review. We will contact leading authors in the field to identify unpublished and ongoing work. We will search the reference list of the systematic reviews relating to FP that have already been identified in the EGM (Ruane‐McAteer et al., 2019). Finally, we will conduct forward citation searching on studies included in the review using Google Scholar.

Searches have been tested and will be conducted with guidance from an information retrieval specialist from the Campbell Collaboration.

The search for qualitative literature will be developed in phase two once the list of included studies and interventions has been compiled. We will then search for qualitative studies or process evaluations relating to included interventions, using a similar approach as outlined above and replacing study design terms with qualitative terms and using more focused terms to search for interventions included in the review.

EPPI Reviewer 4 software will be used for data management, screening, data extraction and appraisal.

#### 5.2.1 Search limits

The search will not be limited by publication status, date or language of production.

#### 5.2.2 Search terms

The search strategy for phase one has been piloted in MEDLINE and detailed search terms and pilot searches are included in Appendix A. Briefly, we will combine search strings using Boolean operator AND for terms relating to family planning AND men/boys. We will combine these with sensitive search filters for study design, adapted from the filter produced by Cochrane EPOC (2017) sample search for quasiexperimental studies. We will apply the LMIC filters developed by Cochrane Effective practice and organisation of care group (EPOC LMIC 2020, v.3). These filters are based on the World Bank list of countries (2019, https://epoc.cochrane.org/lmic-filters). Searches will be tested and adjusted as necessary to account for the unique indexing, field codes and truncation for each database.

Given the very broad range of potential interventions we have decided not to limit the search by intervention terms in the initial stages. We will develop this search string as follows:

1) Search for the combination of the terms for population AND family planning AND study design AND LMIC in two databases (PsycINFO and MEDLINE).
2) Scan the first 200 records retrieved in each database to identify studies that appear to meet our eligibility criteria (400 records screened).
3) We will use this selection of studies to develop and test a comprehensive list of intervention terms.
4) We will then screen a further selection of up to 200 records in each database to identify a new set of up to 20 potentially eligible studies. This new set will then be used to verify that the newly developed string captures the second set of potentially eligible studies.
5) If the search does not capture this second set of potentially eligible studies, the process above will be repeated until we reach saturation of intervention terms. If this process does not improve search specificity without compromising sensitivity, we will revert to searching without adding intervention terms.

We recognise that the intended search combines five search strings, which can result in a less sensitive search. However, given the breadth of the interventions of interest we feel this is necessary to maximise the specificity of the search in order to reduce the number of irrelevant records retrieved.

## 6 DATA COLLECTION AND ANALYSIS

### 6.1 Description of methods used in primary research

The review will include randomised controlled trials and quasiexperimental studies with control groups measuring the effects of programmes engaging men and boys in FP as well as associated process evaluations using quantitative or qualitative methods and any other qualitative research relating to the included experimental studies.

### 6.2 Criteria for determination of independent findings

It is important to ensure that the effects of an individual intervention are only counted once and the following conventions will therefore apply.

- If there are sufficient eligible studies reporting multiple and dependent effect sizes (i.e., occurring in more than 20 eligible studies) then robust variance estimation will be employed to account for dependency in the data. This technique calculates the variance between effect sizes to give the variable of interest a quantifiable standard error. It has been shown to calculate correct results with a minimum of 20–30 individual studies (Hedges et al., 2010), although it performs better with an increased quantity of studies.

If there are fewer than 20 studies:

- Where there are *multiple measures reported for the same outcome*, this will be dealt with by selecting the effect size estimate that (a) employed ITT analysis, (b) is measured using the same or a comparable measurement tool to other include studies.
- Studies with more than one *intervention or control group* will be discussed with the full team of authors to decide if eligible interventions are similar enough to combine and compare as if they are one intervention group (and likewise for multiple control groups). If not, each intervention group will contribute separate effect sizes to the meta‐analysis and the comparator group data will be divided by the number of intervention groups included in the analysis, to avoid double counting of comparator participants.
- In the case of the inclusion of *multiple cohorts of participants* in one study, we will treat each cohort as a separate study contributing a single effect size estimate to the meta‐analysis. If there is a shared control group, the control group sample size will be divided by the number of cohorts included. If different cohorts in a study fall into different subgroups in our meta‐analyses, they will be considered separately in the subgroup analysis and no overall summary of effect will be calculated combining subgroups in those cases.

#### 6.2.1 Selection of studies

Records identified in the searches will be entered into EndNote x9 and duplicates removed. Obviously irrelevant records will be removed by one author (e.g., those that clearly do not relate to implementation of a psychosocial or behavioural intervention, do not contain information or data on male participation, do not relate to FP‐related behaviour change).

The remaining records will then be screened in duplicate by title and abstract by two screeners, working independently, using EPPI Reviewer 4. To ensure quality control, a third reviewer will also screen the first 100 records, chosen at random, and discuss agreements and disagreements with the two screeners and calculate Cohen's *κ* to measure interrater reliability. This process will be repeated to ensure moderate agreement, until Cohen's *κ* reaches 0.41 or above (McHugh, 2012), and the review team are satisfied that screeners are making consistent decisions. We will also make use of tools in EPPI Reviewer 4 to expedite the screening process, including keyword highlighting and AI ranking of studies.

The full text of potentially relevant records will then be retrieved and the screening and quality control process will be repeated as outlined above with a smaller sample of 10 full texts, employing independent dual screening of all records thereafter. Screeners will record reasons for excluding studies at this stage. Any disagreements between screeners will be discussed with a third reviewer until a consensus is reached. If no consensus is reached, the wider team of authors will be consulted and the final decision will be made by ÁA.

#### 6.2.2 Data extraction and management

When eligible studies have been identified, we will undertake dual data extraction, where two people will both complete data extraction and risk of bias assessments independently for each study. Coding, quality and risk of bias assessments will be carried out by trained researchers. Any discrepancies will be discussed with other members of the team of authors until a consensus is reached.

### 6.3 Details of study coding categories

A draft data extraction form is included in Appendix B. This coding framework will be developed and piloted before undertaking data extraction using EPPI Reviewer 4 software. Extraction forms will be based on the principles of “Effectiveness‐plus” reviews to allow more detailed analysis of the causal chain and enable us to answer questions relating to systems and processes. If sufficient detail is lacking, we will contact authors. At a minimum, we will extract the following: publication details, geographical location of study, intervention details including setting, dosage and implementation, delivery personnel, descriptions of the outcomes of interest including instruments used to measure, design and type of trial, sample size of intervention and control groups, data required to calculate Hedge's *g* effect sizes and quality and risk of bias assessment. It is anticipated that we will also extract more detailed information on the interventions such as: when the intervention is delivered, key programme components (as described by study authors). Alongside extracting data on programme components, descriptive information for each of the studies will be extracted and coded to allow for sensitivity and subgroup analysis. This will include information on:

- Study characteristics: design, sample sizes, measures and attrition rates, funder of the study, and whether the study was conducted by a research team associated with the programme or an independent team;
- Stage of programme development, for example, whether it is a new programme being piloted or an established programme being replicated or scaled‐up, trialed in a new location or context and whether or not it has been adapted to fit the new context;
- Intervention details, such as the theory of change, components within the intervention, who delivers and who is the intended recipient of the programme;
- Extent to which the programme was delivered as intended (fidelity);
- Participant demographic variables relating to *PROGRESS Plus* criteria (O'Neill et al., 2014): Place of residence, race, occupation, gender/sex, religion, education, socioeconomic status, social capital, possible discriminatory characteristics, features of relationships, time‐dependent disadvantage; and
- Intervention setting, for example, healthcare setting, schools, community or at home.

Quantitative data will be extracted to allow for calculation of effect sizes (such as mean change scores and standard error, or pre and postmeans and *SD*s). Data will be extracted for the intervention and control groups on the relevant outcomes in order to assess the intervention effects.

#### 6.3.1 Assessment of risk of bias in included studies

Assessment of methodological quality and risk for bias in randomised trials will be conducted using the Cochrane Risk of Bias tool for Randomised Controlled Trials (Higgins et al., 2011). This is a standard tool, which takes the forms of a series of questions about the randomisation procedures and blinding. Nonrandomised studies will be coded using ROBINS‐I (Sterne et al., 2016), qualitative studies coded using Jimenez et al. (2018) critical appraisal tool and quantitative process evaluation studies using the EPPI Centre Tool (EPPI‐Centre, 2003).

#### 6.3.2 Measures of treatment effect

Where outcomes are reported as continuous variables, the main effect size metric to be used in the meta‐analyses will be the standardised mean difference, with its 95% confidence interval. Within this, Hedges' *g* will be used to correct for any small sample bias. Where other effect sizes have been reported (such as Cohen's *d*) these will be converted to Hedges' *g* for the meta‐analysis, using formulae provided in the Cochrane Handbook (Higgins & Green, 2011). Where outcomes are measured as dichotomous, data meta‐analysis will be conducted using odds ratio, with a random effects model (see below).

#### 6.3.3 Unit of analysis issues

If studies involve group‐level allocation, where possible, data will be included which have been adjusted to account for the effects of clustering, typically through the use of multilevel modelling or adjusting estimates using the intra‐cluster correlation coefficient (ICC). Where the effects of clustering have not been taken into account in the report of the study, estimates of effect size will be adjusted following guidance in the Cochrane Handbook (Higgins & Green, 2011). If ICC is not reported, external estimates will be obtained from studies that provide the best match on outcome measures and types of clusters from existing databases of ICCs (Ukoumunne et al., 1999) or other similar studies within the review.

#### 6.3.4 Dealing with missing data

If study reports do not contain sufficient data to allow calculation of effect size estimates, we will contact the original authors to request necessary summary data, such as means and *SD*s or standard errors. If no information is forthcoming, the study cannot be included in the meta‐analysis and will instead be included in a narrative synthesis. Where data are missing due to attrition from the study, studies will be included and sensitivity analysis performed to check the impact of including studies with more than 20% attrition. Where available, results of “intention to treat” analysis will be preferred over “as treated” or “per protocol” analysis in individual studies.

#### 6.3.5 Assessment of heterogeneity

Heterogeneity will be assessed first through visual inspection of the forest plot and checking for overlap of confidence intervals and second through the *Q*, *I* ^2^ and *τ* ^2^ statistics. Investigation of the source of heterogeneity is addressed in data synthesis section.

#### 6.3.6 Assessment of reporting biases

A funnel plot and Egger's linear regression test will be included to check for publication bias across included studies (Sterne & Egger, 2006). Where the funnel plot is asymmetrical, this suggests either publication bias or other bias which relates to smaller studies showing different treatment effects to larger studies. The trim and fill method will be used in a sensitivity analysis where the funnel plot is asymmetrical (Higgins & Green, 2011). This is a nonparametric technique which removes the smaller studies causing irregularity until there is a new symmetrical pooled estimate. The removed studies are then filled back in to assess the robustness of the new estimate.

To ensure robustness of the review and to account for individual studies that appear to exert an undue influence on findings, process sensitivity analysis will also be carried out on domains relating to the quality of the included studies.

#### 6.3.7 Data synthesis

We will adopt a CCA approach to analysis (Kneale et al., 2018). The logic model will inform pairwise analysis to identify which interventions are effective, mediator and moderator analysis to identify the pathways to effectiveness (quantitative CCA), and meta‐regression to assess the impact of specific components and characteristics and combinations of components and characteristics of effective interventions and/or moderation by characteristics of the population/setting. The logic model will be tested using appropriate meta‐analytic techniques, depending on the nature of the relationships or “links” in the causal chain tested (Ivers et al., 2014; Tanner‐Smith & Grant, 2018). Pairwise meta‐analysis is appropriate for establishing overall effectiveness, whereas meta‐regression and/or subgroup and sensitivity analysis provides an opportunity to explore the influence of multiple components of the multiple elements of complex interventions to better understand sources of complexity and their impact on the effect estimates for the interventions of interest, as well as how these components interact with others (Tanner‐Smith & Grant, 2018).

The analytic approach for each of our objectives is outlined below. Further detail on the integration of qualitative evidence is elaborated in the section on qualitative evidence.

1) *What is the nature and extent of experimental evidence on engaging men and boys in FP and what gaps in research knowledge exist?*
  This will be answered through narrative synthesis detailing the geographical spread of studies, the aspects of FP studied, quality of the evidence base and the relative proportions of interventions adopting a gender blind, gender sensitive and GT approach. We will also consider intervention subtypes that emerge from analysis of interventions descriptions/theories of change and also integrate qualitative evidence.
2) *What are the impacts of FP interventions that involve men and boys on FP‐related outcomes?*
  This will be assessed through pairwise meta‐analysis of the effects of these interventions compared to a control condition for each outcome specified. We have selected a range of outcomes along the causal chain.
3) *What are the key components of effective interventions?*
  The key components of interventions will be identified and coded through assessing the study reports alongside any documentation on the development of the intervention/programme and qualitative process evaluations that can provide a deeper understanding of which components of interventions are likely to be essential. We will then quantitatively test the impact of the presence or absence of these components using sub‐group analysis or, if the data allows, meta‐regression.
4) *What characteristics and combinations of characteristics are associated with positive FP‐related outcomes?*
  As above, key characteristics of interventions will be identified, coded and tested using subgroup analysis or, if the data allows, meta‐regression.
5) *Do outcomes vary by context and participant characteristics?*
  This will be assessed through subgroup analysis and investigation of statistical heterogeneity.
6) *Are there any unintended or adverse outcomes?*
  This will be assessed primarily by extracting data on reported adverse effects and conducting pairwise meta‐analysis on common adverse effects, alongside synthesis of qualitative evidence indicating the potential adverse effects.
7) *What are the system‐ and process‐level barriers to and enablers of effective models of FP involving men and boys?*

This will be assessed through examination of the qualitative evidence.

### 6.4 Approach to meta‐analysis

Given the diverse range of interventions that this review is likely to find, random effects models, using inverse‐variance estimation, will be used as the basis for meta‐analysis. The analyses will be conducted using r and the range of commands externally developed to conduct meta‐analysis with r such as meta and metafor and clubSandwich to RVE.

### 6.5 Main effects (Objectives 2 and 6)

The main effects analysis, synthesising the evidence in relation to the effects of FP programmes in general, will be undertaken using the approach to meta‐analysis outlined above for each primary and secondary outcome in turn, with separate analysis for different durations of follow‐up (see *Duration of follow up*).

### 6.6 Sensitivity analysis (Objective 5)

For each outcome, the following sensitivity analyses will be undertaken to assess whether there are potential influences relating to studies that appear to exert an undue influence on findings and based on study quality. We will assess the impact of the inclusion of both randomised trials and quasiexperimental studies, by conducing separate analysis for the randomised trials only. We will also examine the impact of risk of bias by conducting separate analysis omitting studies with an overall rating of high risk of bias.

### 6.7 Subgroup analysis and investigation of heterogeneity (Objectives 3–5)

The complexity of the logic model means that we will undertake a large number of planned subgroup analysis and meta‐regressions to assess the differential effects in relation to the components of interventions, characteristics of the intervention delivery, population of interest and context.

The subgroup analyses (using random‐effects models) will group studies, or subgroups within studies by subcategory and estimate overall effects sizes for each. Subgroup analyses will only be carried out where studies included in the subgroup analysis are sufficiently similar to each other in all other respects, such as whether the interventions delivered to younger and older people are similar enough to be confident that the subgroup analysis reflects differences in the effects for different populations rather than different intervention effects.

### 6.8 Treatment of qualitative research (Objective 7)

As noted, qualitative evidence will be used to inform decision‐making in relation to the quantitative synthesis and we will integrate qualitative and quantitative evidence in order to answer the review questions. The analysis of qualitative data will be informed by the “Best‐Fit” Framework Synthesis approach (Carroll et al., 2011). This method adopts a deductive approach, using an a priori theoretical model to map and code review data (Carroll et al., 2011). Where data are identified that cannot be coded against themes included in the a priori model, thematic analysis is applied to code these data and identify new themes. This approach directs users to revise and iterate the a priori framework to produce a new model consistent with available evidence (Carroll et al., 2013).

The framework for this synthesis is the logic model presented in Figure 1. We will adopt a purposive sampling approach when selecting which qualitative studies to include in our review. We will aim to select studies that relate to one or more of the interventions included in the quantitative synthesis. The purpose of the analysis will be to provide rich evidence on why, for whom and under what circumstances these interventions do or do not work and also to provide evidence on one of more of the “links” in the causal chain outlined in our logic model. If we find more than 20 such studies, we will sample a selection of studies that cover a broad geographical spread and address the broadest range of included interventions. The selection and synthesis of qualitative studies will continue until we have reached saturation in the data.

Qualitative extractions will be coded against the a priori themes from the logic model. Theme headings will be entered into NVivo and data coded deductively under the relevant theme headings. We will also examine the data for evidence that cannot be coded under the a priori themes, with the aim of creating new inductively derived themes. This data will be analysed using Thematic Analysis. We will revisit the evidence to explore the relationships between a priori themes and new themes and their implications for revising the review logic model and we will integrate findings from the quantitative synthesis using a tabular or narrative format. Finally, we will test this synthesis and model by exploring the issues of dissonance and the impact of variables such as quality.

## Footnotes

1 The search conducted for the EGM and review of reviews included a search of Campbell, Cochrane, PROSPERO along with comprehensive searching of academic databases and grey literature sources.

## APPENDIX A INVOLVE_FP SEARCH DEVELOPMENT AND TESTING

The search has been developed and tested in MEDLINE. Searches will be tested and adjusted as necessary to account for the unique indexing, field codes and truncation for each database. In the review, we will report all searches in sufficient detail to allow for replication.

**Population: men and boys**

Men or man or male or males or boy or boys or masculin* or father* or husband.ti,ab,fx,kf,hw.

This search string was adapted from (Ruane‐McAteer et al., 2018) by adding “husband”. The addition of “partner” and gender equality terms was tested but these did not add unique relevant records and added irrelevant records and were removed.

**Condition of interest: Family planning**

Family planning or ((unintended or unwanted or unplanned or planned or wanted or intended) ADJ Pregnan*) or Contracepti* or birth control or adolescent pregnancy or birth spacing or birth interval* or child spacing or pregnancy interval* or delay pregnancy or abortion or abortions).ti,ab,fx,kf,hw.

This string was adapted from Ruane‐McAteer et al. (2018) and Robinson et al. (in process). Each term was tested using Mesh headings to identify relevant overlapping concepts and terms. Each potential term and varitants of the term (e.g., abortion, abort*, abortion or abortions) was tested to ensure it added unique records not captured by other terms using NOT. Only terms that added unique records were included in the final string.

We also discussed and tested the addition of terms relating to fertility and infertility. The team concluded that, as the review is focused on family planning in the sense of preventing unintended pregnancy we decided not to include specific search terms for fertility or infertility. We felt that we could not do justice to considerations around infertility treatment within the context of this review and we believe that it warrants its own review. Particularly because men are so often left out of discourses on fertility which is damaging for both men and women as it shifts focus and responsibility for reproduction, and by extension child rearing, solely to women.

**Study design**

randomized controlled trial or controlled clinical trial or pragmatic clinical trial or multicenter study).pt.

nonrandomized controlled trials as topic/

interrupted time series analysis/

controlled before‐after studies/

(randomis* or randomiz* or randomly).ti,ab.

groups.ab.

(trial or multicenter or multi center or multicentre or multi centre).ti.

(intervention? or effect? or impact? or controlled or control group? or (before adj5 after) or (pre adj5 post) or ((pretest or pre test) and (posttest or post test)) or quasiexperiment* or quasi experiment* or evaluat* or time series or time point? or repeated measur* or ((nonequivalent or non equivalent) adj3 control*)).ti,ab.

(program* and evaluat*).ti,ab,kw.

This string was developed by adapting the sample string produced by Cochrane EPOC (2017) 2 Cochrane Effective Practice and Organisation of Care (EPOC). [Resource title]. EPOC Resources for review authors, 2017. https://epoc.cochrane.org/resources/epoc-resources-review-authors (Accessed June 30, 2020). adding (((nonequivalent or non equivalent) adj3 control$) and (program* and evaluat*).ti,ab,kw which added at least 3 potentially relevant records not otherwise captured.

**Low and middle income countries**

(afghanistan or albania or algeria or american samoa or angola or “antigua and barbuda” or antigua or barbuda or argentina or armenia or armenian or aruba or azerbaijan or bahrain or bangladesh or barbados or republic of belarus or belarus or byelarus or belorussia or byelorussian or belize or british honduras or benin or dahomey or bhutan or bolivia or “bosnia and herzegovina” or bosnia or herzegovina or botswana or bechuanaland or brazil or brasil or bulgaria or burkina faso or burkina fasso or upper volta or burundi or urundi or cabo verde or cape verde or cambodia or kampuchea or khmer republic or cameroon or cameron or cameroun or central african republic or ubangi shari or chad or chile or china or colombia or comoros or comoro islands or iles comores or mayotte or democratic republic of the congo or democratic republic congo or congo or zaire or costa rica or “cote d'ivoire” or “cote d' ivoire” or cote divoire or cote d ivoire or ivory coast or croatia or cuba or cyprus or czech republic or czechoslovakia or djibouti or french somaliland or dominica or dominican republic or ecuador or egypt or united arab republic or el salvador or equatorial guinea or spanish guinea or eritrea or estonia or eswatini or swaziland or ethiopia or fiji or gabon or gabonese republic or gambia or “georgia (republic)” or georgian or ghana or gold coast or gibraltar or greece or grenada or guam or guatemala or guinea or guinea bissau or guyana or british guiana or haiti or hispaniola or honduras or hungary or india or indonesia or timor or iran or iraq or isle of man or jamaica or jordan or kazakhstan or kazakh or kenya or “democratic people's republic of korea” or republic of korea or north korea or south korea or korea or kosovo or kyrgyzstan or kirghizia or kirgizstan or kyrgyz republic or kirghiz or laos or lao pdr or “lao people's democratic republic” or latvia or lebanon or lebanese republic or lesotho or basutoland or liberia or libya or libyan arab jamahiriya or lithuania or macau or macao or “macedonia (republic)” or macedonia or madagascar or malagasy republic or malawi or nyasaland or malaysia or malay federation or malaya federation or maldives or indian ocean islands or indian ocean or mali or malta or micronesia or federated states of micronesia or kiribati or marshall islands or nauru or northern mariana islands or palau or tuvalu or mauritania or mauritius or mexico or moldova or moldovian or mongolia or montenegro or morocco or ifni or mozambique or portuguese east africa or myanmar or burma or namibia or nepal or netherlands antilles or nicaragua or niger or nigeria or oman or muscat or pakistan or panama or papua new guinea or new guinea or paraguay or peru or philippines or philipines or phillipines or phillippines or poland or “polish people's republic” or portugal or portuguese republic or puerto rico or romania or russia or russian federation or ussr or soviet union or union of soviet socialist republics or rwanda or ruanda or samoa or pacific islands or polynesia or samoan islands or navigator island or navigator islands or “sao tome and principe” or saudi arabia or senegal or serbia or seychelles or sierra leone or slovakia or slovak republic or slovenia or melanesia or solomon island or solomon islands or norfolk island or norfolk islands or somalia or south africa or south sudan or sri lanka or ceylon or “saint kitts and nevis” or “st. kitts and nevis” or saint lucia or “st. lucia” or “saint vincent and the grenadines” or saint vincent or “st. vincent” or grenadines or sudan or suriname or surinam or dutch guiana or netherlands guiana or syria or syrian arab republic or tajikistan or tadjikistan or tadzhikistan or tadzhik or tanzania or tanganyika or thailand or siam or timor leste or east timor or togo or togolese republic or tonga or “trinidad and tobago” or trinidad or tobago or tunisia or turkey or “turkey (republic)” or turkmenistan or turkmen or uganda or ukraine or uruguay or uzbekistan or uzbek or vanuatu or new hebrides or venezuela or vietnam or viet nam or middle east or west bank or gaza or palestine or yemen or yugoslavia or zambia or zimbabwe or northern rhodesia or global south or africa south of the sahara or sub‐saharan africa or subsaharan africa or africa, central or central africa or africa, northern or north africa or northern africa or magreb or maghrib or sahara or africa, southern or southern africa or africa, eastern or east africa or eastern africa or africa, western or west africa or western africa or west indies or indian ocean islands or caribbean or central america or latin america or “south and central america” or south america or asia, central or central asia or asia, northern or north asia or northern asia or asia, southeastern or southeastern asia or south eastern asia or southeast asia or south east asia or asia, western or western asia or europe, eastern or east europe or eastern europe or developing country or developing countries or developing nation? or developing population? or developing world or less developed countr* or less developed nation? or less developed population? or less developed world or lesser developed countr* or lesser developed nation? or lesser developed population? or lesser developed world or under developed countr* or under developed nation? or under developed population? or under developed world or underdeveloped countr* or underdeveloped nation? or underdeveloped population? or underdeveloped world or middle income countr* or middle income nation? or middle income population? or low income countr* or low income nation? or low income population? or lower income countr* or lower income nation? or lower income population? or underserved countr* or underserved nation? or underserved population? or underserved world or under served countr* or under served nation? or under served population? or under served world or deprived countr* or deprived nation? or deprived population? or deprived world or poor countr* or poor nation? or poor population? or poor world or poorer countr* or poorer nation? or poorer population? or poorer world or developing econom* or less developed econom* or lesser developed econom* or under developed econom* or underdeveloped econom* or middle income econom* or low income econom* or lower income econom* or low gdp or low gnp or low gross domestic or low gross national or lower gdp or lower gnp or lower gross domestic or lower gross national or lmic or lmics or third world or lami countr* or transitional countr* or emerging economies or emerging nation?).ti,ab,sh,kf.

We used the search string developed and tested by Cochrane EPOC (EPOC LMIC filters 2020 (v.3)) retrieved from https://epoc.cochrane.org/lmic-filters on June 29th, 2020. These filters are based on the World Bank list of countries (2019), classified as low‐income, lower‐middle‐income or upper‐middle‐income economies and were prepared by Cochrane Effective practice and organisation of care group.

**Intervention**

Given the very broad range of potential interventions we have decided not to limit the search by intervention terms in the initial stages. We will develop this search string as follows:

1) Search for the combination of the terms for population AND family planning AND study design AND LMIC in two databases (psych info and medline).
2) Scan the first 200 records retrieved in each database to quickly identify studies that appear to meet our eligibility criteria (400 records screened).
3) We will use this selection of studies to develop and test a comprehensive list of intervention terms.
4) We will then screen a further selection of up to 200 records in each database to identify a new set of up to 20 potentially eligible studies. This new set will then be used to verify that the newly developed string captures the second set of potentially eligible studies.
5) If the search does not capture this second set the process above will be repeated until we reach saturation of intervention terms. If this process does not improve search specificity without compromising sensitivity we will revert to searching without adding intervention terms.

**Pilot search example**

Database(s): **Ovid MEDLINE(R) ALL** 1946 to June 29, 2020

Search Strategy:

**Table:**

#	Searches	Results
1	(Men or man or male or males or boy or boys or masculin* or father* or husband).ti,ab,fx,kf,hw.	8,968,395
2	(Family planning or ((unintended or unwanted or unplanned or planned or wanted or intended) adj Pregnan*) or Contracepti* or birth control or adolescent pregnancy or birth spacing or birth interval* or child spacing or pregnancy interval* or delay pregnancy or abortion or abortions).ti,ab,fx,kf,hw.	195,603
3	(afghanistan or albania or algeria or american samoa or angola or “antigua and barbuda” or antigua or barbuda or argentina or armenia or armenian or aruba or azerbaijan or bahrain or bangladesh or barbados or republic of belarus or belarus or byelarus or belorussia or byelorussian or belize or british honduras or benin or dahomey or bhutan or bolivia or “bosnia and herzegovina” or bosnia or herzegovina or botswana or bechuanaland or brazil or brasil or bulgaria or burkina faso or burkina fasso or upper volta or burundi or urundi or cabo verde or cape verde or cambodia or kampuchea or khmer republic or cameroon or cameron or cameroun or central african republic or ubangi shari or chad or chile or china or colombia or comoros or comoro islands or iles comores or mayotte or democratic republic of the congo or democratic republic congo or congo or zaire or costa rica or “cote d'ivoire” or “cote d' ivoire” or cote divoire or cote d ivoire or ivory coast or croatia or cuba or cyprus or czech republic or czechoslovakia or djibouti or french somaliland or dominica or dominican republic or ecuador or egypt or united arab republic or el salvador or equatorial guinea or spanish guinea or eritrea or estonia or eswatini or swaziland or ethiopia or fiji or gabon or gabonese republic or gambia or “georgia (republic)” or georgian or ghana or gold coast or gibraltar or greece or grenada or guam or guatemala or guinea or guinea bissau or guyana or british guiana or haiti or hispaniola or honduras or hungary or india or indonesia or timor or iran or iraq or isle of man or jamaica or jordan or kazakhstan or kazakh or kenya or “democratic people's republic of korea” or republic of korea or north korea or south korea or korea or kosovo or kyrgyzstan or kirghizia or kirgizstan or kyrgyz republic or kirghiz or laos or lao pdr or “lao people's democratic republic” or latvia or lebanon or lebanese republic or lesotho or basutoland or liberia or libya or libyan arab jamahiriya or lithuania or macau or macao or “macedonia (republic)” or macedonia or madagascar or malagasy republic or malawi or nyasaland or malaysia or malay federation or malaya federation or maldives or indian ocean islands or indian ocean or mali or malta or micronesia or federated states of micronesia or kiribati or marshall islands or nauru or northern mariana islands or palau or tuvalu or mauritania or mauritius or mexico or moldova or moldovian or mongolia or montenegro or morocco or ifni or mozambique or portuguese east africa or myanmar or burma or namibia or nepal or netherlands antilles or nicaragua or niger or nigeria or oman or muscat or pakistan or panama or papua new guinea or new guinea or paraguay or peru or philippines or philipines or phillipines or phillippines or poland or “polish people's republic” or portugal or portuguese republic or puerto rico or romania or russia or russian federation or ussr or soviet union or union of soviet socialist republics or rwanda or ruanda or samoa or pacific islands or polynesia or samoan islands or navigator island or navigator islands or “sao tome and principe” or saudi arabia or senegal or serbia or seychelles or sierra leone or slovakia or slovak republic or slovenia or melanesia or solomon island or solomon islands or norfolk island or norfolk islands or somalia or south africa or south sudan or sri lanka or ceylon or “saint kitts and nevis” or “st. kitts and nevis” or saint lucia or “st. lucia” or “saint vincent and the grenadines” or saint vincent or “st. vincent” or grenadines or sudan or suriname or surinam or dutch guiana or netherlands guiana or syria or syrian arab republic or tajikistan or tadjikistan or tadzhikistan or tadzhik or tanzania or tanganyika or thailand or siam or timor leste or east timor or togo or togolese republic or tonga or “trinidad and tobago” or trinidad or tobago or tunisia or turkey or “turkey (republic)” or turkmenistan or turkmen or uganda or ukraine or uruguay or uzbekistan or uzbek or vanuatu or new hebrides or venezuela or vietnam or viet nam or middle east or west bank or gaza or palestine or yemen or yugoslavia or zambia or zimbabwe or northern rhodesia or global south or africa south of the sahara or sub‐saharan africa or subsaharan africa or africa, central or central africa or africa, northern or north africa or northern africa or magreb or maghrib or sahara or africa, southern or southern africa or africa, eastern or east africa or eastern africa or africa, western or west africa or western africa or west indies or indian ocean islands or caribbean or central america or latin america or “south and central america” or south america or asia, central or central asia or asia, northern or north asia or northern asia or asia, southeastern or southeastern asia or south eastern asia or southeast asia or south east asia or asia, western or western asia or europe, eastern or east europe or eastern europe or developing country or developing countries or developing nation? or developing population? or developing world or less developed countr* or less developed nation? or less developed population? or less developed world or lesser developed countr* or lesser developed nation? or lesser developed population? or lesser developed world or under developed countr* or under developed nation? or under developed population? or under developed world or underdeveloped countr* or underdeveloped nation? or underdeveloped population? or underdeveloped world or middle income countr* or middle income nation? or middle income population? or low income countr* or low income nation? or low income population? or lower income countr* or lower income nation? or lower income population? or underserved countr* or underserved nation? or underserved population? or underserved world or under served countr* or under served nation? or under served population? or under served world or deprived countr* or deprived nation? or deprived population? or deprived world or poor countr* or poor nation? or poor population? or poor world or poorer countr* or poorer nation? or poorer population? or poorer world or developing econom* or less developed econom* or lesser developed econom* or under developed econom* or underdeveloped econom* or middle income econom* or low income econom* or lower income econom* or low gdp or low gnp or low gross domestic or low gross national or lower gdp or lower gnp or lower gross domestic or lower gross national or lmic or lmics or third world or lami countr* or transitional countr* or emerging economies or emerging nation?).ti,ab,sh,kf.	1,911,880
4	1 and 2 and 3	11,501
5	(randomized controlled trial or controlled clinical trial or pragmatic clinical trial or multicenter study).pt.	785,447
6	nonrandomized controlled trials as topic/	704
7	interrupted time series analysis/	892
8	controlled before‐after studies/	520
9	(randomis* or randomiz* or randomly).ti,ab.	901,958
10	groups.ab.	2,062,983
11	(trial or multicenter or multi center or multicentre or multi centre).ti.	262,437
12	(intervention? or effect? or impact? or controlled or control group? or (before adj5 after) or (pre adj5 post) or ((pretest or pre test) and (posttest or post test)) or quasiexperiment* or quasi experiment* or evaluat* or time series or time point? or repeated measur* or ((nonequivalent or non equivalent) adj3 control*)).ti,ab.	9,655,893
13	(program* and evaluat*).ti,ab,kw.	197,643
14	5 or 6 or 7 or 8 or 9 or 10 or 11 or 12 or 13	10,757,634
15	exp Animals/	23,265,764
16	Humans/	18,554,106
17	15 not 16	4,711,658
18	14 not 17	8,678,188
19	4 and 18	5492

## APPENDIX B INVOLVE_FP REVIEW DRAFT DATA EXTRACTION FORM

**Table:**

#	Category	Question	Type
	Study Characteristics	Authors	Text
	Study Characteristics	Publication Title	Text
	Study Characteristics	Publication Year	Text
	Study Characteristics	Publication Type (Published; Grey Literature)	Checkbox
	Study Characteristics	Funder	Text
	Study Characteristics	Study Design (RCT, QE, Qualitative Study)	Checkbox
	Study Characteristics	Study/Intervention Aim	Text
	Study Characteristics	Country of Implementation	Text
	Study Characteristics	Sample Size (Intervention Group)	Text
	Study Characteristics	Sample Size (Control Group)	Text
	Study Characteristics	Sample Characteristics	Text
	Study Characteristics	Stage of Programme Development/Evaluation (Pilot, Scale‐up, Established, Transfer, Adaptation)	Checkbox, Info
	Intervention Characteristics	Details of Intervention Inputs (Time and Cost; Implementers; Intervention Materials; Participant and Volunteer Incentives)	Text
	Intervention Characteristics	Intervention Components Coded (Gender; Information; Skills; Problem‐Solving; Social Support; Incentives; Media; Environmental Context and Resources)	Multiple Checkbox
	Intervention Characteristics	Intervention Name	Checkbox, Info
	Intervention Characteristics	Intervention Intended Goal & Rationale	Checkbox, Info
	Intervention Characteristics	Theory(ies) Applied	Checkbox, Info
	Intervention Characteristics	Intervention Design	Checkbox, Info
	Intervention Characteristics	Materials and Procedure	Checkbox, Info
	Intervention Characteristics	Who Provided	Checkbox, Info
	Intervention Characteristics	Who Received	Checkbox, Info
	Place of residence Race Occupation Gender Religion Education Socio‐economic Status Social capital Possible discriminatory characteristics Features of relationships Time‐dependent disadvantage		
	Intervention Characteristics	Mode of Delivery	Checkbox, Info
	Intervention Characteristics	Delivery Setting	Checkbox, Info
	Intervention Characteristics	Delivery Dosage	Checkbox, Info
	Intervention Characteristics	Details of any Tailoring	Checkbox, Info
	Intervention Characteristics	Details of any Modification	Checkbox, Info
	Intervention Characteristics	Details of any Fidelity Assessment	Checkbox, Info
	Moderators	Individual Level Moderators Analysed (Age; Sex; Ethnicity; Disability; Gender identity; Sexual Orientation; Religion; Religiosity; Relationship Status; Reproductive History; Past FP behaviours; Co‐Residency; Urban/Rural Residence; Migrant Status; Attitude and Beliefs about FP; Perceived Gender and Cultural Norms; Attitudes about Sexual Pleasure; Socioeconomic factors; OTHER)	Multiple Checkbox, Info
	Moderators	External Moderators Analysed (Gender Norms; Cultural Norms; Religious Norms; Political and Economic Climate; Legal Context; Current Health Policy and Strategies; Health Systems and Services; FP Supply Chain; Conflict; Disaster; Disease Outbreak; Climate‐Stressed Environment; OTHER)	Multiple Checkbox, Info
	Moderators	Process Metrics Analysed (Intervention Acceptability; Intervention Cost; Intervention Sustainability; Quality of Delivery; Provider Preparedness; Participant Recruitment and Retention; Study Design and Characteristics)	Multiple Checkbox, Info
	Outcomes	Measures Used	Text
	Outcomes	Individual Level Outcomes, Males and Females (Gender Equitable Attitudes; Knowledge related to FP; Attitudes related to FP; Skills related to FP; Contraceptive Uptake and Use; Support for Female Partner Contraceptive Uptake and Use; Delayed Pregnancy; Birth Spacing and Limiting; Support for Male Involvement in FP, *Other*)	Outcome Code
	Outcomes	Interpersonal Level Outcomes, Males and Females (Communication and Quality of Relationships; Equitable Relationships and FP Decision‐making, *Other*)	Outcome Code
	Outcomes	Community Level Outcomes, Males and Females (Wider Community Gender Equitable Attitudes; Support from Family Members, Peers, and Wider Community, *Other*)	Outcome Code
	Outcomes	Service/Organisation Level Outcomes, Males and Females (Service Provider and Educator's Gender Equitable Attitudes; Provision and Polices Involving Males; Quality and Accessibility of FP Services for Males, *Other*)	Outcome Code
	Outcomes	Structural Level Outcomes, Males and Females (Policy Support for Gender Equality; Policy Support for Male Involvement in FP; Resources for FP Provision, *Other*)	Outcome Code
	Impact	Distal Outcomes/Impacts Measured (Gender Quality for Females; Health and Well‐being; Female Agency; Male Trust in Health Services; Male Engagement in Child Birth and Parenting; Quality of Life; Rate of Contraceptive Use, Rate of Unintended Pregnancy; Maternal and Child Morbidity and Mortality; Rate of Unsafe Abortion; Child Marriage; Adolescent Fertility Rate, Average Family Size; HIV and STI Prevalence, Rates of Sexual and Gender‐Based Violence; Health Service Burden; Health Economy Associated with FP Intervention)	Multiple Checkbox, Info
